# Prevalence of BRD-Related Viral Pathogens in the Upper Respiratory Tract of Swiss Veal Calves

**DOI:** 10.3390/ani11071940

**Published:** 2021-06-29

**Authors:** Eveline Studer, Lutz Schönecker, Mireille Meylan, Dimitri Stucki, Ronald Dijkman, Melle Holwerda, Anna Glaus, Jens Becker

**Affiliations:** 1 Clinic for Ruminants, Vetsuisse Faculty, University of Bern, Bremgartenstrasse 109a, 3012 Bern, Switzerland; eveline.studer@vetsuisse.unibe.ch (E.S.); lutz.schoenecker@vetsuisse.unibe.ch (L.S.); mireille.meylan@vetsuisse.unibe.ch (M.M.); dimitri.stucki@vetsuisse.unibe.ch (D.S.); 2 Institute of Veterinary Bacteriology, Vetsuisse Faculty, University of Bern, Länggassstrasse 122, 3001 Bern, Switzerland; 3 Institute of Virology and Immunology, Vetsuisse Faculty, University of Bern, Länggassstrasse 122, 3001 Bern, Switzerland; ronald.dijkman@ifik.unibe.ch (R.D.); melle.holwerda@dbmr.unibe.ch (M.H.); anna.glaus@gmail.com (A.G.); 4 Department of Infectious Diseases and Pathobiology, Vetsuisse Faculty, University of Bern, Länggassstrasse 122, 3012 Bern, Switzerland; 5 Institute for Infectious Diseases, University of Bern, Friedbühlstrasse 51, 3001 Bern, Switzerland; 6 Graduate School for Cellular and Biomedical Science, University of Bern, Mittelstrasse 43, 3012 Bern, Switzerland

**Keywords:** bovine respiratory disease, bovine coronavirus, risk factors, prevalence, calf fattening

## Abstract

**Simple Summary:**

In bovines, infection of the respiratory tract is frequent. It may lead to severe disease and death, and often requires antimicrobial treatment. Antimicrobial treatment is known to be related to antimicrobial resistance, which poses a threat to public health; therefore, efforts to better understand the causes of airway infection must be undertaken. Many different pathogens may be responsible for disease onset, including viruses. The aim of this study was to describe the prevalence of respiratory viruses in Swiss veal calves and to identify risk factors for infection. In a convenience sample of 764 swabs, prevalence rates were determined for bovine respiratory syncytial virus (BRSV, 2.1%), bovine parainfluenza-3 virus (BPI3V, 3.3%), bovine coronavirus (BCoV, 53.5%), influenza D virus (IDV, 4.1%), and influenza C virus (ICV, 0%). Due to the high prevalence rate, risk factors were investigated for BCoV. Younger calves tested positive more frequently than older calves. Increasing group size was associated with increasing probability for occurrence of BCoV. To summarize, different viral pathogens circulate in veal calves.

**Abstract:**

The prevention of bovine respiratory disease is important, as it may lead to impaired welfare, economic losses, and considerable antimicrobial use, which can be associated with antimicrobial resistance. The aim of this study was to describe the prevalence of respiratory viruses and to identify risk factors for their occurrence. A convenience sample of 764 deep nasopharyngeal swab samples from veal calves was screened by PCR for bovine respiratory syncytial virus (BRSV), bovine parainfluenza-3 virus (BPI3V), bovine coronavirus (BCoV), influenza D virus (IDV), and influenza C virus (ICV). The following prevalence rates were observed: BRSV, 2.1%; BPI3V, 3.3%; BCoV, 53.5%; IDV, 4.1%; ICV, 0%. Logistic mixed regression models were built for BCoV to explore associations with calf management and housing. Positive swab samples were more frequent in younger calves than older calves (>100 days; *p* < 0.001). The probability of detecting BCoV increased with increasing group size in young calves. Findings from this study suggested that young calves should be fattened in small groups to limit the risk of occurrence of BCoV, although an extended spectrum of risk factors for viral associated respiratory disorders such as nutritional aspects should be considered in future studies.

## 1. Introduction

Since the 1960s, bovine respiratory disease (BRD) has been a major challenge for the beef and dairy industry, particularly in veal calf farming, as well as for veterinarians and researchers. Clinical BRD may require the application of antimicrobial drugs, increased labor, and decreased welfare [1,2,3]. Additionally, reduced profitability due to decreased performance and death is frequent [4]. Veal calf mortality has been estimated at approximately 2.9–5.1% in Europe [5,6,7,8]. In veal and beef production, performance and profitability can be quantified, e.g., through weight loss or reduced average daily gain in the living animal, and through carcass traits after slaughter. In a previous study, BRD decreased average daily gain and had significant effects on hot carcass weight and marbling scores in feedlot cattle [9]. In veal production, calves that experienced a BRD episode showed significant short-term weight loss, reductions in hot carcass weight, and lower fat cover at slaughter [10]. Assuming that weight loss leads to anorexia, depression, a febrile response, and inflammation, this results in concerns for animal welfare in addition to the economic aspects. These circumstances emphasize the need for a better understanding of the etiopathogenesis of BRD and improved disease control strategies.

Etiologically, BRD is known as a multifactorial process that includes the triad of environmental components, host factors, and various microorganisms that can lead to a severe respiratory syndrome [11,12]. The viral pathogens involved include bovine respiratory syncytial virus (BRSV), bovine parainfluenza-3 (BPI3V), bovine coronavirus (BCoV), bovine herpesvirus type 1 (BHV-1), and bovine viral diarrhea virus (BVDV) [13,14,15,16,17]. Influenza C and D viruses (ICV and IDV) have emerged in the past decade as additional pathogens in the disease complex [18,19,20]. Viral infections may pave the way for secondary bacterial infection, mostly involving commensal bacteria such as pasteurella and mycoplasma species, among others (e.g., by causing respiratory cilia lining dysfunction and modifying the host immune response) [12,21,22,23].

Pro- or metaphylactic antimicrobial treatment is commonly applied in veal calf herds to reduce morbidity and mortality [1,24]. In Switzerland, however, provision of antimicrobial substances for prophylactic purposes has been banned since 2016 [25]. In the EU, prophylactic use of antimicrobials will be banned by January 2022, based on a new regulation on veterinary medicinal products and medicated feed [26]. Denmark, Finland, Iceland, Norway, Sweden, and the Netherlands have already ended prophylactic group treatments, whereas globally only first attempts have often been made to phase out the use of antibiotics in livestock for prophylactic purposes, often based on action plans from the World Health Organization and the Food and Agriculture Organization of the United Nations [27,28].

Metaphylactic antimicrobial treatment is applied in Swiss veal calf herds and worldwide to reduce morbidity and mortality [5,22], and has been observed to be successful in decreasing the incidence of BRD [29,30]. These treatments may contribute to an increased selection pressure on pathogenic and commensal bacteria [24,31]. Increasing antimicrobial resistance (AMR) is an issue of global importance, which emphasizes that antimicrobials should be used according to prudent use guidelines [32] and that antimicrobial use (AMU) should eventually be drastically reduced. As viral infections can promote bacterial infection, reducing the risks for viral infection may not only improve animal health but may also indirectly contribute to decreasing AMU and AMR.

Through investigations we conducted on different aspects of veal calf health, we gained further insight into the challenges, etiology, and multifactorial processes involved in BRD, AMU, and AMR in Swiss veal calves [1,6,8,33]. Based on existent nasopharyngeal swabs used in previous projects, the principal aims of the present study were to describe the occurrence of viral pathogens with a potential involvement in BRD in the upper respiratory tract of veal calves in Switzerland (i.e., BRSV, BPI3V, BCoV, ICV, and IDV), to perform a risk factor analysis to explore associations between the presence of these pathogens and factors related to calf management and housing, and to investigate co-infections of viruses and bacteria. Exploration and diminution of risk factors may contribute to avoiding the onset of BRD. If disease is not preventable at the individual or population level, knowledge about prevalent pathogens may help to avoid inappropriate therapy. This study focused on the investigation of the prevalence of viral pathogens in Swiss veal farms and risk factors involved in BRD, although the population under study was limited in size and geographical extension.

## 2. Materials and Methods

### 2.1. Farm Data and Sample Selection

Farms were enrolled within the framework of a veal calf health research collaborative study supervised by one of the authors (M.M.). Within the framework of this collaborative study, extensive data on veal calf transport, barn climate, management and housing, AMU, AMR in bacteria of the respiratory and digestive tract, bacterial resistance to disinfectants, viral pathogens of the respiratory and digestive tract, animal welfare, and economics were collected [8,33,34,35,36,37,38,39]. Farmers were not enrolled randomly but participated voluntarily, leading to a convenience sample of nasopharyngeal swab samples of Swiss veal calves. A total of 4492 calves were enrolled, from which a total of 6138 nasopharyngeal swabs were taken. At the time of the beginning of the screening for viral pathogens in these swabs, a total of 3820 were available, as a subset was no longer available. Samples were stored in a database and sorted by farm ID, animal ID (ear tag number), and collection date. Data collection procedures during field work were determined to collect suitable data for specific research questions [8,33]. Correspondingly, calves were swab-sampled varying numbers of times. To avoid double inclusion of calves as far as possible for the present investigation and to avoid exceeding the necessary number of samples for this prevalence and risk factor analysis, every fifth database entry was retrieved. When selecting samples, we adhered to the recommended minimum of 15 observations per estimated parameter (i.e., risk factor) [40].

All procedures were approved by the competent Committee for Animal Welfare and Protection (authorization numbers BE 63/16 and BE 71/16). Farms were family-run, with a median annual production amount of 64 calves, in addition to the production of commercial milk. Farm characteristics are shown in Table 1. Farmers participated in one of two field data collection programs, which were both run for one year in parallel. Programs were conducted to collect data for various research areas as stated above and were of two different types. The first program was designed as a prospective cohort study to monitor veal calf farms regarding effects of transport, management factors, and barn climate on calf health [8]. Furthermore, it was designed to estimate the prevalence of opportunistic bacterial pathogens associated with BRD [41] and to elicit possible associations of AMU and AMR [36]. The second program was designed as a prospective intervention study, whereby farms implementing a novel concept for veal calf fattening were monitored. These farms were compared to farms not implementing any measure, which served as the control [33].

### 2.2. Sample and Data Collection

A total of 764 samples from 33 farms were included in the virological analyses conducted in this study. Individual identification numbers of the calves were registered during sampling to retrieve animal-level information (e.g., date of birth) from the national animal movement database (Tierverkehrsdatenbank, TVD). Swabs originated from 705 individuals. Swabbing of calves was done as described elsewhere [33]. Briefly, after disinfection of the calves’ nostrils using gauze (Provet AG/Henry Schein Animal Health, Lyssach, Switzerland) soaked in 70% propylalcohol (F25-A Feinsprit 2% MEK, Alcosuisse AG, Bern, Switzerland), the nasopharyngeal epithelium was swabbed using sterile swabs (COPAN Italia SpA, Brescia, Italy). Swabs were immediately transferred into liquid Amies transportation medium (Axonlab SwabAX, liquid Amies, Axon Lab AG, Baden, Switzerland) and transported to the laboratory within 48 h of collection without cooling, whereby bacterial culture was started upon arrival. Detailed data on the prevalence of bacterial pathogens in the nasopharynx of the calves included in this study are presented elsewhere [41]. After preparation of bacteriological cultures, the swabs were cryopreserved at −80 °C for later virological analysis.

In addition to on-farm sample collection, questionnaires were completed with the farmers to collect farm-level information on management practices. The barn capacity was defined as the maximum number of calves that could be fattened at the same time on a respective farm. The number of birth farms was evaluated in two different ways for those farms purchasing calves for fattening: as the total number of suppliers per farm and as the number of birth farms per ten purchased calves as a standardized and comparable measure. Antimicrobial treatment incidence was calculated at the farm-level as TI_DDD_ in treatment days per calf and year based on defined daily doses according to the European Medicines Agency [42,43]. Farmers were asked whether the milk from their dairy cows was provided to the calves whenever somatic cells were elevated according to a positive California mastitis test result (CMT; positivity was defined as a CMT score 2 or higher) [44]. Calves were mainly fed through automated feeding machines providing the same milk for all calves. As the occurrence of elevated somatic cell counts in milk is fluctuant in farms, farmers who provided CMT-positive milk to calves at least once during the study period were classified as implementing this practice.

**Table 1:** Farm characteristics of 33 veal fattening farms in Switzerland. The categories with the corresponding numbers of farms and percentages are indicated for categorical variables. The median, quantiles (25th, 75th), and interquartile range (IQR) values at the farm-level are indicated for continuous variables.

Categorical Variables	Category	Number	[%]	
Stocking routine	continuous	16	48.5	
	all-in-all-out	17	51.5	
Physical contact among calves of neighboring pens	Yes	18	54.5	
	No	15	45.5	
Shared air space among pens	Yes	10	30.3	
	No	23	69.7	
Refusing sick calves upon arrival ^1^	Yes	20	64.5	
	No	11	35.5	
Disinfection of pens after use ^1^	Yes	11	35.5	
	No	20	64.5	
Early slaughter before reaching farm’s average slaughter weight ^1^	Yes	7	21.8	
	No	25	78.1	
Access to outdoor pen	Yes	27	81.8	
	No	6	18.2	
Feeding of CMT-positive milk ^1,2^	Yes	21	65.6	
	No	11	34.3	
Vaccination against respiratory pathogens ^1,3^	Yes	24	77.4	
	No	7	22.5	
**Continuous Variables**	**Median**	**25th Quantile**	**75th Quantile**	**IQR**
Number of calves fattened annually	63.8	43.2	119	75.8
Maximum number of calves per pen	20	10	25	15
Maximum number of calves per barn (barn capacity)	31.5	24.8	46.5	21.8
Total number of birth farms	15.5	10	23.3	13.3
Number of birth farms per ten purchased calves	3.3	2.1	5.5	3.4
Median duration of the fattening period (days)	114	108	123	15
Median average daily weight gain (kg live weight/day)	1.3	1.3	1.4	0.1
Median carcass weight (kg)	128.7	124.2	132.3	8.1
Mean farm mortality (%)	5.1	3.2	7.4	4.2
Median TI_DDD_ (farm-level treatment days per calf and year)	17.1	5.2	36.5	31.3
Purchased calves (%)	94.1	64.3	100	35.7
Median age at purchase (days)	33	29	35	6
Median age at sampling (days)	107	87	130	43
Unwanted early slaughter ^4^ (%)	0	0	0	0

^1^ Information was not available for all farms. ^2^ CMT: California mastitis test, positive = score 2 or higher [44]. ^3^ Bovilis^®^ Bovigrip, MSD Animal Health, Lucerne, CH or Rispoval^®^ RS/Rispoval^®^ RS +PI3, Zoetis, Delémont, CH. ^4^ Defined as slaughter after a fattening period of >70 days [5].

### 2.3. Sample Analyses

Viral RNA was extracted and used as template for real-time reverse transcription polymerase chain reaction (qRT-PCR), as described by elsewhere. Briefly, viral RNA spiked with in-vitro-transcribed eGFP mRNA transcript as an internal control was extracted, then subsequently purified and used as a template in the respective qRT-PCR assay. The primers and probes used for the detection of BRSV, BPIV3, BCoV, ICV, and IDV have been described elsewhere [45,46,47,48,49]. The detection format was set to dual-color hydrolysis probe/UPL probe, FAM, and VIC/HEX. The results were analyzed with the LightCycler^®^ 480 software (version 1.5.1, Roche Diagnostics) using the Abs Quant 2nd derivative max calculation method. The qRT-PCR results for IDV were obtained from a previous study (data not shown). Ct values <35 were considered positive, those between 35 and <45 were classified as weakly positive, and ≥45 as negative. Weakly positive results were included in the virus-positive category for each virus for statistical analyses. Twelve samples were excluded due to defective PCR tests.

### 2.4. Statistical Analyses

Statistical analyses were carried out in R (version 3.6.3, R Core Team 2020, R Foundation for Statistical Computing, Vienna, Austria) using the dplyr, caret, glmnet, gpairs, reshape2, and lme4 packages. Potential risk factors for the presence of BCoV included in the analysis are shown in Table 2. Among the continuous independent variables, the distributions were mostly not normal, with no straightforward approach for transformation. The bimodally distributed continuous variable ‘age at sampling’ was dichotomized into two levels (younger calves: ≤100 days; older calves: >100 days) representing similar numbers of calves. We performed variable selection to find a small number of variables that best reflected the variance of BCoV among the sampled animals. Only the best-fitting variables were then analyzed in a multivariate logistic regression model to describe the relationship of these variables with the occurrence of BCoV in more detail. At first, redundant features were eliminated via Spearman’s rank correlations to reduce the impacts of collinearity. For this, in an intermediate step, the correlation matrix for all continuous variables was calculated using pairwise complete cases. All correlations with an absolute Spearman’s rank coefficient >0.7 were assessed and the variables with higher mean absolute correlations were removed, i.e., the variables ‘calves fattened annually’, ‘barn capacity’, ‘mean group size’, and ‘carcass weight’. The same process was performed with categorical variables, resulting in the elimination of the variable ‘stocking routine’. As the number of remaining variables still exceeded the supported number of parameters in a multivariate model, as mentioned above, we performed a variable selection to reduce the number of variables to a small number of those showing a promising relationship to the outcome variable BCoV. Variable selection procedures based on univariable models and *p*-values have attracted criticism in recent years [50]; therefore, we used the concept of the repeated elastic net technique (RENT) [51]. This technique assumes that multiple models fitted to subsets of the entire data can provide information about the consistency of effects found within the data.

The elastic net model was trained using the caret package, with a tune length of five values for alpha and five values for lambda and with 10-fold cross validation to optimize the hyperparameters (α and λ). We recorded the model specificity (and sensitivity), which was the number of times the model correctly predicted a positive (and negative) BCoV case in the validation set, as well as model accuracy (number of times the overall prediction was correct). In addition, parameter estimates for each variable were extracted. This process was repeated 100 times with a different random subset of data for training each time, and the sensitivity, specificity, accuracy, and parameter estimates were recorded for each repetition. Subsequently, we calculated the mean, median, and 95% confidence intervals for the recorded values. As also suggested by Jenul et al. [51], we calculated the number of times each variable had a non-zero parameter estimate among all 100 repetitions and assessed the 95% confidence interval for the respective parameter estimates. This process does not provide inform regarding statistical significance.

Because the RENT algorithm cannot incorporate random effects, it is possible that the selected variables reflected farm effects. To account for this, we then used a mixed logistic regression model with a binomial distribution for the selected variables ‘age at sampling’, ‘mean group size’ (mean number of calves per pen), and ‘TI_DDD_’ with the BCoV incidence. For simplicity, we performed a separate model for ‘mean group size’ and ‘TI_DDD_’. Each model included the ‘age at sampling’, as well as the interactions with ‘age at sampling’, because three-way interactions including multiple continuous variables are not easy to interpret. To account for farm and animal effects, individual calf identification nested within farms was added as a random intercept term.

## 3. Results

Farm characteristics are shown in Table 1. Overall, BCoV alone and in combination with other viruses was most prevalent in the nasopharynx of the veal calves under study (Table 3). In total, 47.5% of the swab samples were positive for BCoV (ct-range 11.25–34.97) and an additional 6% were weakly positive (ct-range 35–37.53); 2.1% tested positive for BRSV (ct-range 21.55–34.91) and 2.3% for BPI3V (ct-range 21.89–34.62), with an additional 1% of samples testing weakly positive for BPI3V (ct-range 35.01–37.3). Regarding IDV, 4.1% of samples tested positive (ct-range 14.8–34.66). None of the samples tested positive for ICV, while the positive control was valid. Calves positive for BCoV had a median age of 59 days (IQR = 44–114), those positive for BRSV had a median age of 66 days (IQR = 48–125), those positive for BPI3V had a median age of 69 days (IQR = 46–87), and those positive for IDV had a median age of 60 days (IQR = 44–103). The viral pathogens most frequently observed in coinfection with BCoV were IDV (2.9%), BPI3V (2.1%), and BRSV (1.3%; Table 3).

Based on the low prevalence of BRSV, BPI3V, ICV, and IDV in this study, risk factor analysis was performed for BCoV only.

**Figure 1:**
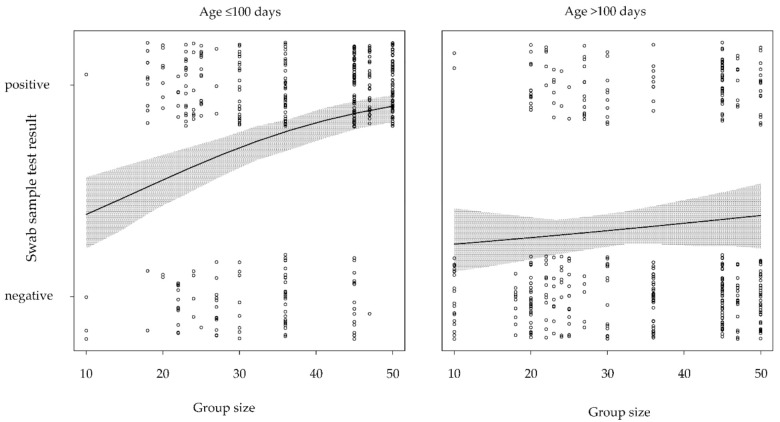
Probability of positive nasopharyngeal swab sample of bovine coronavirus, dependent of mean group size and age.

The prevalence of positive BCoV samples was more than 2-fold higher in younger calves (76 ± 2.3% samples positive, ≤100 days of age) in comparison to older calves (34 ± 2.4% samples positive, >100 days of age) at the farm-level. The elastic net feature selection revealed that three variables (‘age at sampling’, ‘mean group size’, and ‘TI_DDD_’) were selected in more than 50 of the 100 trained models (Table 4). In the logistic regression model, the presence of BCoV in young calves was positively associated with the mean group size (*p* < 0.001; Table 5, Figure 1).

**Figure 2:**
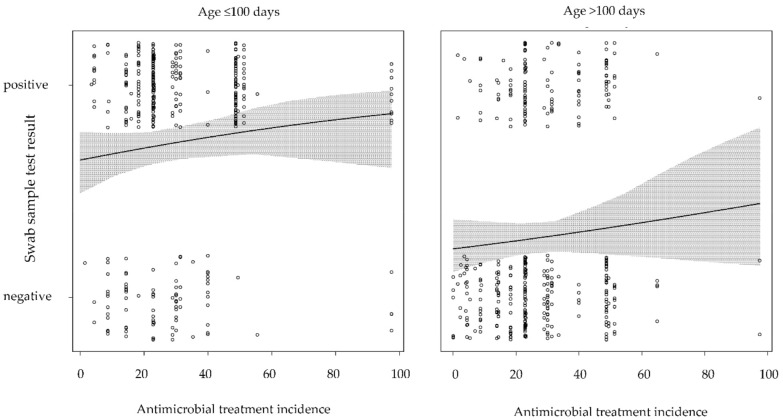
Probability of positive nasopharyngeal swab sample of bovine coronavirus, dependent on antimicrobial treatment incidence and age.

In older calves (>100 days), the presence of BCoV was not associated with the mean group size (*p* = 0.230; Table 5, Figure 1). The presence of BCoV was not associated with TI_DDD_ in younger or older calves (*p* = 0.149 and *p* = 0.253, respectively; Table 6, Figure 2). Antimicrobial treatment incidence (TI_DDD_) was selected in the variable selection process but was not associated with the presence of BCoV in the final logistic regression model for younger or older calves. The number of swabs positive for BCoV that did not test positive for any other virus under study or any of the common bacteria associated with BRD was distinctly higher than the number of BCoV-positive swabs also positive for other organisms (Table 7). Viruses with low prevalence rates (BRSV, BPI3V, IDV) were equally distributed over both age groups (>100 days vs. ≤100 days of age).

## 4. Discussion

The prevalence of respiratory viruses in Swiss veal calves was low in the present study, except for BCoV, which was detected in 53.5% of the samples; thus, only BCoV was included in the risk factor analysis.

Bovine respiratory syncytial virus is recognized as an important pathogen in the BRD complex [52,53,54,55]. Infection with BRSV may be either asymptomatic, limited to the upper airways, or involve both the upper and lower respiratory tract, occasionally causing major clinical signs, suggesting that the host immune response plays a major role in the pathogenesis of BRSV infection [56]. It causes loss of ciliated epithelial cells and predisposes affected airways to secondary bacterial infection [53,57]. We consider the observed prevalence of BRSV in nasopharyngeal swabs of veal calves in our study to be low (2.1%), although comparable studies based on nasopharyngeal swabs are scarce. One study in 231 Limousine beef steers of 6–10 months of age in good nutritional condition detected a BRSV prevalence rate of 14.2% [58].

Similarly, the prevalence of BPI3V was low (3.3%). The exact role of BPI3V in the pathogenesis of BRD is still unclear [59,60]. Sampling acutely diseased calves revealed the presence of BPI3V in 7% (nasal swabs) and 8.1% (broncho-alveolar lavage) of the samples, respectively [16,61].

The low prevalence rates of BRSV and BPI3V may in part be attributable to the immune status of the calves, as, although vaccination against BRSV and BPI3V is generally poorly implemented in Swiss veal fattening farms, it was observed to be performed on 49–84% of the farms and 55% of the calves in other studies respectively [1,8]. The low observed prevalence rates of BRSV and BPI3V, the two respiratory viruses against which vaccines are available for calves in Switzerland, raises the question of whether vaccination can provide targeted protection with the pathogens included in the vaccines, a question that cannot be answered based on the present results, but should be addressed in future studies.

Alternatively, these results may indicate that BRSV and BPI3V are not widespread in veal calf populations outside of BRD outbreaks. They may infect an individual calf transiently and possibly initiate pathologic processes, which are subsequently perpetuated by bacterial pathogens [60].

Influenza D virus was first described in swine [62], but soon after cattle were suggested to be the reservoir species for this virus [63]. Both influenza C and influenza D viruses have now been shown to be associated with BRD in North America [20,49,64]. In the Swiss veal calf population under study, IDV was present at a prevalence rate of 4.1%. This was in accordance with other European studies describing IDV in cattle [18,65]. In contrast, none of our samples tested positive for ICV. Influenza C virus had been identified in US cattle with BRD before (4.2% of 1525 nasal swab and lung tissue specimens [49]), but to our knowledge not yet on the European continent.

Since Switzerland is officially free from BHV-1 and more than 99% of cattle husbandries are recognized as officially free from BVDV [66], we did not test for these viruses in this study.

Bovine coronavirus is the possible causative agent of three distinct clinical syndromes in cattle: calf diarrhea, winter dysentery in adults, and respiratory infections in cattle of various ages, including the BRD complex [55,67,68,69]; however, the role of BCoV as a primary BRD pathogen remains controversial. It was detected by PCR assays or virus isolation in several studies in which cattle displaying signs of respiratory disease were sampled by nasal swabbing [68,70,71,72]. In two studies where nasal swabs from calves and feedlot cattle in acute outbreaks of respiratory disease were analyzed, it was the most prevalent pathogen (22.9% and 24.7%, respectively) [61,64]; however, it has also been detected in nasal swabs from clinically healthy calves and fattening steers [58,72]. In another study where nasal swabs of Canadian dairy calves were investigated for respiratory pathogens and their relationships with clinical status, lung consolidation, and average daily gain, BCoV was not associated with clinical score or lung consolidation using transthoracic ultrasonography [73]. Ellis (2019) suggested that BCoV gains significance in respiratory disease only in interplay with other microorganisms [74]. In addition, concurrent pathogens, viral load, and immunosuppression are potential cofactors that can exacerbate the severity of BCoV infection [68,70]. In our study, calves were not sampled during outbreaks of respiratory disease and detailed information on the health status of the individuals at the time of sampling was not recorded. Taken together with the high prevalence observed in this study, our results suggest that BCoV is common in the veal calf population.

In our study, BCoV prevalence was higher in younger calves than older calves. Potentially, this was due to virus transmission during crowding and commingling after purchase. Additionally, at a young age, the calves’ immune response may be insufficient to eliminate viruses efficiently. This suggests that most calves may eventually eliminate BCoV with advancing age.

A larger mean group size was associated with an increased probability for the presence of BCoV in younger calves. This is in line with other findings, as increasing herd size has been described as a risk factor for the detection of BCoV [75,76]. In contrast, a larger mean group size was not associated with an increased probability of the presence of BCoV in older calves. Compared to BRSV and BPI3V, BCoV may persist longer in the nasopharynx due to its possibly lower pathogenicity or reduced stimulation of the immune system, leading to a higher prevalence. Impaired animal health may be a confounding factor influencing both the TI_DDD_ and the presence of BCoV. No significant association was observed between these two parameters. In this study, TI_DDD_ was investigated as an indirect indicator of animal health because our dataset did not allow for stratification by direct indicators of animal health (such as cough or nasal discharge). Antimicrobial treatment incidences of the selected farms were in line with previous findings [33,41], and treatments were administered due to the suboptimal health status of the calves or as metaphylaxis to prevent the spread of disease. However, the validity of TI_DDD_ as a health indicator has been questioned, as it may partly be influence by the farmers’ previous experiences and routines, especially regarding metaphylactic treatment [8].

For the assessment of potential risk factors for BCoV infection, we measured a total of 33 variables. As a single statistical model would overfit using all variables simultaneously, we first performed variable selection to find a small number of candidate variables which showed a consistent association with BCoV infection within our data. With the RENT algorithm, we chose a novel approach for variable selection, which improves upon some of the criticism addressed towards the standard method of performing multiple univariable models and choosing the variables based on *p*-values. Firstly, RENT uses penalized regression, which allows modelling of a large number of variables at once without overfitting, thereby taking confounding effects among the variables into account. As a result, the selected variables not only reflect direct univariable relationships, but also include important confounders. Secondly, by modeling multiple iterations with only a random subset of the data each, RENT selects variables with a consistent effect in all or most portions of the data, rather than variables with a strong effect. This reduces the likelihood of selecting variables that show an association by chance. Thirdly, the selection of the variables is based on model fit and not on *p*-values; therefore, false discoveries due to multiple testing against a fixed significance level are minimal. Only the 95% confidence intervals are subject to an increased false discovery rate; however, since the major selection criterion is the amount of subsets in which a variable shows an association with the outcome, the impact of biased confidence intervals is negligible.

The detection of BCoV was not associated with the detection of other viral respiratory pathogens in the present study. This finding is in line with the study by Pardon et al. (2020) [16]. In that study, detection of *M. haemolytica* was associated with the detection of BCoV in bronchoalveolar lavage samples from calves [16]. We did not find such an association between virus and bacteria in nasopharyngeal samples; however, the prevalence data on bacterial pathogens were generated using cultures (in contrast, Pardon et al. (2020) used PCR testing [16]), whereas our outcome regarding viruses was generated using PCR for our risk factor analysis [41]. This mix of methods may have influenced our results. Finally, the population included in the study was not a representative random sample of the Swiss veal calf population, but it allowed for a first exploration of the prevalence of respiratory viruses, as no data at all have been available in Switzerland to date. To conclude, the prevalence of respiratory viruses in Swiss veal calves was low in the present study, except for BCoV. Despite the fact that the role of BCoV as a primary pathogen in BRD is controversial, we evaluated the relevant risk factors for BCoV in the present study, revealing ‘age at sampling’ and ‘mean group size’ in younger calves (≤100 days of age) as being significantly associated with the presence of BCoV. Apparently, young calves in larger groups are particularly likely to harbor BCoV. Increasing group size has been shown to be a risk factor for increased mortality, morbidity (BRD), and reduced growth rate in veal and dairy calves [1,77,78]. We selected a variety of relevant possible risk factors for our study; however, many more possible risk factors exist, such as climate-related parameters, hygiene, and nutrition. Regarding nutrition, interest is often focused on the colostral phase; however, regarding the plane of nutrition after the colostral phase, controversial results exist. Overly nutritious diets may be able to exacerbate bacterial disease [79] and calves fed greater crude protein diets might have an increased likelihood of being diagnosed with fever associated with BRD [80,81]. Both scenarios might be found in intense animal production systems. Controversially, others have found that low planes of nutrition negatively influence the responses to pathogen challenges [82]. Calf nutrition and its effects on BRD were not within the focus of the present study.

## 5. Conclusions

In conclusion, the results of the present study show that increased group size may be associated with occurrence of BRD-related viral pathogens, in our case BCoV. This is supported by the findings of other studies, where increasing group size was also associated with increased risk for the occurrence of bacterial pathogens related to BRD [41]. The practical advice for farmers and veterinarians is to fatten calves in small groups until 100 days of age, although the pathogenicity of viruses involved in the BRD complex, in our study particularly of the frequently isolated BCoV, has not been clarified. Further research on BRD is needed and should include an extended spectrum of possible risk factors.

## Figures and Tables

**Table 2:** Potential risk factors for positive swab sample test for bovine coronavirus (BCoV) in Swiss veal calves from 33 farms.

Level	Category	Description
Potential farm-level risk factors	Management	Number of calves fattened annually; mean group size; stocking routine (continuous/all-in-all-out); barn capacity ^1^; physical contact among calves of neighboring pens (yes/no); shared air space among pens (yes/no); total number of birth farms ^2^; number of birth farms per ten purchased calves; refusing sick calves upon arrival (yes/no); median duration of the fattening period; median average daily weight gain; median carcass weight; slaughter weight; disinfection of pens after use (yes/no); farm-level mortality rate; unwanted early slaughter (yes/no); access to outdoor pen (yes/no); feeding of CMT-positive milk (yes/no) ^3^; vaccination against respiratory pathogens (yes/no); TI_DDD_^4^
Potential individual calf level risk factors	Sampled calves	Purchased calf ^5^ (yes/no); age at purchase (days); age at sampling (days) ^6^; death before slaughter ^7^ (yes/no);
	Detection of respiratory viruses ^8^	Positive test for BRSV, BPI3V, IDV, or ICV (yes/no)
	Detection of respiratory bacteria by culture	Positive culture for *Histophilus somni*, Bisgaard Taxon 39, *Mannheimia haemolytica*, *Pasteurella multocida*, *Mycoplasma bovis*, or *Mycoplasma dispar* (yes/no)

^1^ Barn capacity: number of calves that can be fattened at the same time on one farm. ^2^ Total number of farms selling calves to respective veal calf farm. ^3^ CMT: California mastitis test, score 2 or higher [44]. ^4^ Antimicrobial treatment incidence rates in defined daily doses per animal/year (ddd/ay; EMA, 2013, 2016). ^5^ In contrast to calves born at the fattening farm. ^6^ Dichotomized according to bimodal distribution at ≤100 days and >100 days of age at sampling. ^7^ Calf succumbed to disease before slaughter (i.e., mortality on individual level; yes/no). ^8^ BRSV: bovine respiratory syncytial virus; BPI3V: bovine parainfluenza 3 virus; IDV: influenza D virus; ICV: influenza C virus.

**Table 3:** Overall prevalence and farm-level prevalence (including mean, standard deviation, minimum, and maximum values) of respiratory pathogens BCoV, BRSV, BPI3V, ICV, and IDV in Swiss veal calves from 33 fattening farms, as well as overall prevalence of swab samples positive for more than one respiratory virus (based on 764 nasopharygeal swab samples).

Virus ^1^	Number of Positive Samples ^2^	Overall Prevalence	Mean Farm-level Prevalence	Standard Deviation	Min.	Max.
BCoV	402	53.5	23.2	28.5	0	100
BRSV	16	2.1	1.3	4.2	0	23.1
BPI3V	25	3.3	0.9	2.24	0	9.3
ICV	0	0	0	0	0	0
IDV	31	4.1	2.3	8.9	0	50
Coinfections						
BCOV + IDV	22	2.9				
BCOV + BRSV	10	1.3				
BCOV + BPI3V	16	2.1				
BCOV + IDV + BPI3V	4	0.5				
BCOV + IDV + BRSV	1	0.0 ^3^				
IDV + BPI3V	5	0.0 ^3^				
IDV + BRSV	2	0.0 ^3^				

^1^ Prevalence rates are shown separately for each virus. Additionally, coinfections are also shown separately. ^2^ Number of swabs that tested positive (ct <35) or weakly positive (ct between 35 and <45) for the respective virus or combination. ^3^ Mathematically rounded.

**Table 4:** Selection criteria from the elastic net variable selection algorithm from 100 iterations for variables associated with the detection of bovine coronavirus (BCoV) from nasopharygeal swab samples, as well as quality measurements of the selection process. Variables that occurred in more than 50 models and had a 95% confidence interval not crossing zero were selected for a final inferential model.

Variable	Number of Models	Mean	Median	Lower 95% CI	Upper 95% CI
Age at sampling	100	−0.62	−0.57	−1.03	−0.42
Mean group size	81	0.08	0.08	0.00	0.24
TI_DDD_^1^	64	0.07	0.02	0.00	0.50
Physical contact among calves of neighbouring pens	48	−0.07	0.00	−0.52	0.00
Death before slaughter ^2^	43	0.07	0.00	0.00	0.55
Feeding CMT-positive milk	40	−0.05	0.00	−0.36	0.00
Positive culture for *Pasteurella multocida*	36	−0.04	0.00	−0.25	0.00
Total number of birth farms per veal farm ^3^	32	−0.03	0.00	−0.36	0.00
Disinfection of pen after use	30	−0.04	0.00	−0.26	0.00
Positive culture for *Histophilus somni*	24	0.02	0.00	0.00	0.24
BRSV-positive swab sample	23	0.03	0.00	0.00	0.30
Average daily weight gain	22	0.04	0.00	0.00	0.44
Positive culture for *Mycoplasma bovis*	22	0.02	0.00	0.26	0.00
Positive culture for Bisgaard taxon 39	21	−0.02	0.00	0.00	−0.16
Shared airspace between pens	20	0.02	0.00	0.00	0.27
IDV-positive swab sample	18	0.02	0.00	0.00	0.25
Purchased calf	17	−0.01	0.00	0.00	−0.14
Positive culture for *Mycoplasma dispar*	17	0.01	0.00	0.13	0.00
Access to outdoor pen	16	−0.04	0.00	0.00	−0.42
Age at purchase	16	0.00	0.00	−0.10	0.04
Positive culture for *Mannheimia haemolytica*	15	0.00	0.00	−0.05	0.09
BPI3V-positive swab sample	14	−0.01	0.00	−0.13	0.05
Refusing sick calves upon arrival	11	−0.03	0.00	0.00	−0.39
Vaccination against respiratory pathogens	10	−0.01	0.00	0.00	−0.22
Early slaughter before reaching farm’s average slaughter weight	9	−0.01	0.00	0.00	−0.16
Duration of the fattening period	8	0.00	0.00	0.00	0.04
Mortality	7	0.00	0.00	−0.06	0.02
Number of birth farms per ten purchased calves	5	0.00	0.00	0.00	0.02
Quality measurements					
Accuracy		0.69	0.69	0.64	0.73
Sensitivity		0.69	0.70	0.57	0.76
Specificity		0.69	0.69	0.63	0.74

^1^ Farm-level antimicrobial treatment incidence of defined daily doses per animal/year (ddd/ay; EMA, 2013, 2016). ^2^ Calf succumbed to disease before slaughter (i.e., mortality on individual calf level; yes/no). ^3^ Total number of farms selling calves to respective veal calf farm.

**Table 5:** Results from logistic mixed regression analysis for the detection of BCoV ^1^ in nasopharyngeal swab samples in 33 Swiss veal calf farms regarding group size ^2^ and age ^3^, as well as the interaction between group size and age. Additionally, the regression slope ^4^ for group size is provided for each age class separately.

Parameter	Category	Estimate	Standard Error	*z*-Value	*p*-Value
Intercept		−1.14	0.55	−2.07	0.039
Group size ^2^ Age ^3^		0.07	0.02	4.01	<0.001
	>100 days	−0.16	0.57	−0.27	0.786
Group size: Age	>100 days	−0.05	0.02	−3.20	0.001
Regression slope for each age class ^4^					
Younger	≤100 days	0.07	0.02	4.01	<0.001
Older	>100 days	0.02	0.01	1.20	0.230

^1^ Bovine coronavirus. ^2^ Mean farm-level group size. ^3^ Dichotomized at >100 days according to bimodal distribution. ^4^ Increase in BCoV-positive swab samples (%) with increasing mean group size.

**Table 6:** Results from logistic mixed regression for the detection of BCoV ^1^ in nasopharyngeal swab samples from 33 Swiss veal calf farms regarding TI_DDD_^2^ and age ^3^, as well as the interaction between antimicrobial treatment incidence and age. Additionally, the regression slope ^4^ for antimicrobial treatment incidence (i.e., the increase in BCoV prevalence with increasing daily dosage) is provided for each age ^3^ class separately.

Parameter	Category	Estimate	Standard Error	*z*-Value	*p*-Value
Intercept		0.62	0.33	1.89	0.059
TI_DDD_^2^ Age ^3^		0.01	0.01	1.44	0.149
	>100 days	−1.88	0.35	−5.41	<0.001
TI_DDD_: Age	>100 days	−0.003	0.01	−0.27	0.788
					
Regression slope for each age class ^4^					
Younger	≤100 days	0.01	0.01	1.44	0.149
Older	>100 days	0.01	0.01	1.44	0.253

^1^ Bovine coronavirus. ^2^ Antimicrobial treatment incidence of defined daily doses per animal-year (ddd/ay; EMA, 2013, 2016). ^3^ Dichotomized at 100 days according to bimodal distribution. ^4^ Increase in BCoV-positive swab samples (%) with increasing TI_DDD_.

**Table 7:** Prevalence of respiratory viruses ^1^ and bacteria ^2^ in 384 ^3^ deep nasopharyngeal swab samples from calves from 12 Swiss veal calf farms. Only bacteria found in combination in more than five 5 observations are presented.

Organism	BCoV	BRSV	BPI3V	ICV	IDV ^4^	BCoV + BRSV	BCoV + BPI3V	BCoV + IDV	BCoV + BRSV +IDV	BCoV + BPI3V + IDV	IDV + BPI3V	IDV + BRSV
Virus isolation only	117	2	2		3	7	3	3		1	1	
*P. multocida*	49		1				1	7		1		
*M. dispar*	34				2					1		
*M. pernigra*	14		1		1		2	1				
*M. bovis*	12					1						
*M. haemolytica*	9						1					

^1^ Virus detection by polymerase chain reaction. ^2^ Isolation of bacteria (Pasteurella (P.) multocida, Mannheimia (M.) haemolytica, Histophilus (H.) somni, Mannheimia (M.) pernigra, Mycoplasma (M.) bovis, Mycoplasma (M.) dispar) by culture as described in [41]. ^3^ Full dataset including both viral and bacterial analyses was available for 657 out of a total of 764 samples included in this study. Out of these 657 samples, 384 tested positive for at least one of the viral pathogens analyzed in this study, 273 tested negative for all viruses. BCoV: bovine coronavirus; BRSV: bovine respiratory syncytial virus; BPI3V: bovine parainfluenza-3 virus; ICV: influenza C virus; ^4^ IDV: influenza D virus.

## Data Availability

The data presented in this study are available on request from the corresponding author.

## References

[1] Lava M., Schüpbach-Regula G., Steiner A., Meylan M. (2016). Antimicrobial drug use and risk factors associated with treatment incidence and mortality in Swiss veal calves reared under improved welfare conditions. Prev. Vet. Med..

[2] Dubrovsky S.A., Van Eenennaam A.L., Aly S.S., Karle B.M., Rossitto P.V., Overton M.W., Lehenbauer T.W., Fadel J.G. (2020). Preweaning cost of bovine respiratory disease (BRD) and cost-benefit of implementation of preventative measures in calves on California dairies: The BRD 10K study. J. Dairy Sci..

[3] Bull E.M., Bartram D.J., Cock B., Odeyemi I., Main D.C.J. (2021). Construction of a conceptual framework for assessment of health-related quality of life in calves with respiratory disease. Animal.

[4] Becker J., Steiner A., Meylan M., Hauser B., Straub U. (2021). Vergleichende Wirtschaftlichkeitsanalyse des Kälbermastsystems «Freiluftkalb» und der konventionellen IP-SUISSE-Labelmast. Schweiz. Arch. Tierheilkd..

[5] Bähler C., Steiner A., Luginbühl A., Ewy A., Posthaus H., Strabel D., Kaufmann T., Regula G. (2012). Risk factors for death and unwanted early slaughter in Swiss veal calves kept at a specific animal welfare standard. Res. Vet. Sci..

[6] Lava M., Pardon B., Schüpbach-Regula G., Keckeis K., Deprez P., Steiner A., Meylan M. (2016). Effect of calf purchase and other herd-level risk factors on mortality, unwanted early slaughter, and use of antimicrobial group treatments in Swiss veal calf operations. Prev. Vet. Med..

[7] Bokma J., Boone R., Deprez P., Pardon B. (2019). Risk factors for antimicrobial use in veal calves and the association with mortality. J. Dairy Sci..

[8] Schnyder P., Schönecker L., Schüpbach-Regula G., Meylan M. (2019). Effects of management practices, animal transport and barn climate on animal health and antimicrobial use in Swiss veal calf operations. Prev. Vet. Med..

[9] Schneider M.J., Tait R.G., Busby W.D., Reecy J.M. (2009). An evaluation of bovine respiratory disease complex in feedlot cattle: Impact on performance and carcass traits using treatment records and lung lesion scores. J. Anim. Sci..

[10] Pardon B., Hostens M., Duchateau L., Dewulf J., De Bleecker K., Deprez P. (2013). Impact of respiratory disease, diarrhea, otitis and arthritis on mortality and carcass traits in white veal calves. BMC Vet. Res..

[11] Apley M. (2006). Bovine Respiratory Disease: Pathogenesis, Clinical Signs, and Treatment in Lightweight Calves. Vet. Clin. N. Am. Food Anim. Pract..

[12] Confer A.W. (2009). Update on bacterial pathogenesis in BRD. Anim. Heath Res. Rev..

[13] Härtel H., Nikunen S., Neuvonen E., Tanskanen R., Kivelä S.-L., Aho P., Soveri T., Saloniemi H. (2004). Viral and Bacterial Pathogens in Bovine Respiratory Disease in Finland. Acta Vet. Scand..

[14] Hägglund S., Svensson C., Emanuelson U., Valarcher J.F., Alenius S. (2006). Dynamics of virus infections involved in the bovine respiratory disease complex in Swedish dairy herds. Vet. J..

[15] Pardon B., De Bleecker K., Dewulf J., Callens J., Boyen F., Catry B., Deprez P. (2011). Prevalence of respiratory pathogens in diseased, non-vaccinated, routinely medicated veal calves. Vet. Rec..

[16] Pardon B., Callens J., Maris J., Allais L., Van Praet W., Deprez P., Ribbens S. (2020). Pathogen-specific risk factors in acute outbreaks of respiratory disease in calves. J. Dairy Sci..

[17] Grissett G.P., White B.J., Larson R.L. (2015). Structured Literature Review of Responses of Cattle to Viral and Bacterial Pathogens Causing Bovine Respiratory Disease Complex. J. Vet. Intern. Med..

[18] Ducatez M.F., Pelletier C., Meyer G. (2015). Influenza D virus in cattle, France, 2011–2014. Emerg. Infect. Dis..

[19] Ferguson L., Olivier A.K., Genova S., Epperson W.B., Smith D.R., Schneider L., Barton K., McCuan K., Webby R.J., Wan X.-F. (2016). Pathogenesis of Influenza D Virus in Cattle. J. Virol..

[20] Nissly R.H., Zaman N., Ibrahim P.A.S., McDaniel K., Lim L., Kiser J.N., Bird I., Chothe S.K., Bhushan G.L., Vandegrift K. (2020). Influenza C and D viral load in cattle correlates with bovine respiratory disease (BRD): Emerging role of orthomyxoviruses in the pathogenesis of BRD. Virology.

[21] Griffin D., Chengappa M.M., Kuszak J., McVey D.S. (2010). Bacterial pathogens of the bovine respiratory disease complex. Vet. Clin. N. Am. Food Anim. Pract..

[22] Caswell J.L. (2014). Failure of Respiratory Defenses in the Pathogenesis of Bacterial Pneumonia of Cattle. Vet. Pathol..

[23] Guterbock W.M. (2014). The impact of BRD: The current dairy experience. Anim. Heath Res. Rev..

[24] Pardon B., Catry B., Dewulf J., Persoons D., Hostens M., De Bleecker K., Deprez P. (2012). Prospective study on quantitative and qualitative antimicrobial and anti-inflammatory drug use in white veal calves. J. Antimicrob. Chemother..

[25] Schweizer Bundesrat Verordnung über die Tierarzneimittel (Tierarzneimittelverordnung, TAMV). https://www.fedlex.admin.ch/eli/cc/2004/592/de.

[26] European Commisson (2019). Implementation of Regulation (EU) 2019/6 on Veterinary Medicinal Products and Regulation (EU) 2019/4 on Medicated Feed.

[27] World Health Organisation WHO Guidelines on Use of Medically Important Antimicrobials in Food-Producing Animals. https://www.who.int/foodsafety/areas_work/antimicrobial-resistance/cia_guidelines/en/#:~:text=WHO.

[28] Food and Agruculture Organization of the United Nations The FAO Action Plan on Antimicrobial Resistance 2016–2020|Global Forum on Food Security and Nutrition (FSN Forum). http://www.fao.org/fsnforum/resources/fsn-resources/fao-action-plan-antimicrobial-resistance-2016-2020.

[29] Lofgreen G.P. (1983). Mass Medication in Reducing Shipping Fever-Bovine Respiratory Disease Complex in Highly Stressed Calves. J. Anim. Sci..

[30] Nickell J.S., White B.J. (2010). Metaphylactic antimicrobial therapy for bovine respiratory disease in stocker and feedlot cattle. Vet. Clin. N. Am. Food Anim. Pract..

[31] Jarrige N., Cazeau G., Morignat E., Chanteperdrix M., Gay E. (2017). Quantitative and qualitative analysis of antimicrobial usage in white veal calves in France. Prev. Vet. Med..

[32] World Health Organisation (WHO) (2014). Antimicrobial Resistance: Global Report on Surveillance. https://apps.who.int/iris/handle/10665/112642?locale-attribute=zh&show=full.

[33] Becker J., Schüpbach-Regula G., Steiner A., Perreten V., Wüthrich D., Hausherr A. (2020). Effects of the novel concept ‘outdoor veal calf’ on antimicrobial use, mortality and weight gain in Switzerland. Prev. Vet. Med..

[34] Kauer R., Koch M.C., Schönecker L., Becker J., Holwerda M., Glaus A., Hierweger M., Werder S., Dijkman R., Meylan M. (2020). Fecal Shedding of Bovine Astrovirus CH13/NeuroS1 in Veal Calves. J. Clin. Microbiol..

[35] Schnyder P., Schönecker L., Schüpbach-Regula G., Meylan M. (2019). Transporte von Mastkälbern vom Geburts- auf den Mastbetrieb und Kälbermanagement in Schweizer Geburtsbetrieben. Schweiz. Arch. Tierheilkd..

[36] Schönecker L., Schnyder P., Overesch G., Schüpbach-Regula G., Meylan M. (2019). Associations between antimicrobial treatment modalities and antimicrobial susceptibility in Pasteurellaceae and E. coli isolated from veal calves under field conditions. Vet. Microbiol..

[37] Hausherr A., Becker J., Meylan M., Wüthrich D., Collaud A., Rossano A., Perreten V. (2019). Antibiotic and quaternary ammonium compound resistance in Escherichia coli from calves at the beginning of the fattening period in Switzerland (2017). Schweiz Arch Tierheilkd.

[38] Moser L., Becker J., Schüpbach-regula G., Kiener S., Grieder S., Keil N., Hillmann E., Steiner A., Meylan M. (2020). Welfare assessment in calves fattened according to the “outdoor veal calf” concept and in conventional veal fattening operations in switzerland. Animals.

[39] Wüthrich D., Brilhante M., Hausherr A., Becker J., Meylan M., Perreten V. (2019). Correction for Wüthrich et al., “A Novel Trimethoprim Resistance Gene, dfrA35, Characterized from Escherichia coli from Calves”. mSphere.

[40] Austin P.C., Steyerberg E.W. (2017). Events per variable (EPV) and the relative performance of different strategies for estimating the out-of-sample validity of logistic regression models. Stat. Methods Med. Res..

[41] Schönecker L., Schnyder P., Schüpbach-Regula G., Meylan M., Overesch G. (2020). Prevalence and antimicrobial resistance of opportunistic pathogens associated with bovine respiratory disease isolated from nasopharyngeal swabs of veal calves in Switzerland. Prev. Vet. Med..

[42] EMA Revised ESVAC Reflection Paper on Collecting Data on Consumption of Antimicrobial Agents Per Animal Species, on Technical Units of Measurement and Indicators for Reporting Consumption of Antimicrobial Agents in Animals. https://www.ema.europa.eu/en/documents/scientific-guideline/revised-european-surveillance-veterinary-antimicrobial-consumption-esvac-reflection-paper-collecting_en.pdf.

[43] EMA Defined Daily Doses for Animals (DDDvet) and Defined Course Doses for Animals (DCDvet): European Surveillance of Veterinary Antimicrobial Consumption (ESVAC). http://www.ema.europa.eu/docs/en_GB/document_library/Other/2016/04/WC500205410.pdf.

[44] Kandeel S.A., Morin D.E., Calloway C.D., Constable P.D. (2018). Association of California Mastitis Test Scores with Intramammary Infection Status in Lactating Dairy Cows Admitted to a Veterinary Teaching Hospital. J. Vet. Intern. Med..

[45] Boxus M., Letellier C., Kerkhofs P. (2005). Real Time RT-PCR for the detection and quantitation of bovine respiratory syncytial virus. J. Virol. Methods.

[46] Horwood P.F., Mahony T.J. (2011). Multiplex real-time RT-PCR detection of three viruses associated with the bovine respiratory disease complex. J. Virol. Methods.

[47] Kishimoto M., Tsuchiaka S., Rahpaya S.S., Hasebe A., Otsu K., Sugimura S., Kobayashi S., Komatsu N., Nagai M., Omatsu T. (2017). Development of a one-run real-time PCR detection system for pathogens associated with bovine respiratory disease complex. J. Vet. Med. Sci..

[48] Holwerda M., Kelly J., Laloli L., Stürmer I., Portmann J., Stalder H., Dijkman R. (2019). Determining the replication kinetics and cellular tropism of influenza D virus on primary well-differentiated human airway epithelial cells. Viruses.

[49] Zhang H., Porter E., Lohman M., Lu N., Peddireddi L., Hanzlicek G., Marthaler D., Liu X., Bai J. (2018). Influenza C virus in cattle with respiratory disease, United States, 2016–2018. Emerg. Infect. Dis..

[50] Heinze G., Dunkler D. (2017). Five myths about variable selection. Transpl. Int..

[51] Jenul A., Schrunner S., Liland K.H., Indahl U.G., Futsaether C.M., Tomic O. (2020). RENT—Repeated Elastic Net Technique for Feature Selection. arXiv.

[52] Baker J.C., Ellis J.A., Clark E.G. (1997). Bovine respiratory syncytial virus. Vet. Clin. N. Am. Food Anim. Pract..

[53] Ellis J.A. (2009). Update on viral pathogenesis in BRD. Anim. Health Res. Rev..

[54] Brodersen B.W. (2010). Bovine respiratory syncytial virus. Vet. Clin. N. Am. Food Anim. Pract..

[55] MacLachlan N.J., Dubovi E.J. (2017). Fenner’s Veterinary Virology.

[56] Valarcher J.-F., Taylor G. (2007). Bovine respiratory syncytial virus infection. Vet. Res..

[57] Sudaryatma P.E., Nakamura K., Mekata H., Sekiguchi S., Kubo M., Kobayashi I., Subangkit M., Goto Y., Okabayashi T. (2018). Bovine respiratory syncytial virus infection enhances Pasteurella multocida adherence on respiratory epithelial cells. Vet. Microbiol..

[58] Pratelli A., Cirone F., Capozza P., Trotta A., Corrente M., Balestrieri A., Buonavoglia C. (2021). Bovine respiratory disease in beef calves supported long transport stress: An epidemiological study and strategies for control and prevention. Res. Vet. Sci..

[59] Ellis J.A. (2010). Bovine Parainfluenza-3 Virus. Vet. Clin. N. Am. Food Anim. Pract..

[60] Fern M., Ferreras C., Javier F., Benavides J. (2020). Production Significance of Bovine Respiratory Disease Lesions in Slaughtered Beef Cattle. Animals.

[61] O’Neill R., Mooney J., Connaghan E., Furphy C., Graham D.A. (2014). Patterns of detection of respiratory viruses in nasal swabs from calves in Ireland: A retrospective study. Vet. Rec..

[62] Hause B.M., Ducatez M., Collin E.A., Ran Z., Liu R., Sheng Z., Armien A., Kaplan B., Chakravarty S., Hoppe A.D. (2013). Isolation of a Novel Swine Influenza Virus from Oklahoma in 2011 Which Is Distantly Related to Human Influenza C Viruses. PLoS Pathog..

[63] Ferguson L., Eckard L., Epperson W.B., Long L.P., Smith D., Huston C., Genova S., Webby R., Wan X.F. (2015). Influenza D virus infection in Mississippi beef cattle. Virology.

[64] Mitra N., Cernicchiaro N., Torres S., Li F., Hause B.M. (2016). Metagenomic characterization of the virome associated with bovine respiratory disease in feedlot cattle identified novel viruses and suggests an etiologic role for influenza D virus. J. Gen. Virol..

[65] Snoeck C.J., Oliva J., Pauly M., Losch S., Wildschutz F., Muller C.P., Hübschen J.M., Ducatez M.F. (2018). Influenza D virus circulation in cattle and swine, Luxembourg, 2012–2016. Emerg. Infect. Dis..

[66] Bundesamt für Lebensmittelsicherheit und Veterinärwesen Ausrottung BVD. https://www.blv.admin.ch/blv/de/home/tiere/tierseuchen/bekaempfung/ausrottung-bvd.html.

[67] Clark M.A. (1993). Bovine Coronavirus. Br. Vet. J..

[68] Storz J., Lin X., Purdy C.W., Chouljenko V.N., Kousoulas K.G., Enright F.M., Gilmore W.C., Briggs R.E., Loan R.W. (2000). Coronavirus and Pasteurella infections in bovine shipping fever pneumonia and Evans’ criteria for causation. J. Clin. Microbiol..

[69] Hodnik J.J., Ježek J., Starič J. (2020). Coronaviruses in cattle. Trop. Anim. Health Prod..

[70] Decaro N., Campolo M., Desario C., Cirone F., D’Abramo M., Lorusso E., Greco G., Mari V., Colaianni M.L., Elia G. (2008). Respiratory disease associated with bovine coronavirus infection in cattle herds in Southern Italy. J. Vet. Diagn. Investig..

[71] Paller T., Hostnik P., Pogacnik M., Toplak I. (2017). The prevalence of ten pathogens detected by a real-time PCR method in nasal swab samples collected from live cattle with respiratory disease. Slov. Vet. Res..

[72] Workman A.M., Kuehn L.A., McDaneld T.G., Clawson M.L., Loy J.D. (2019). Longitudinal study of humoral immunity to bovine coronavirus, virus shedding, and treatment for bovine respiratory disease in pre-weaned beef calves. BMC Vet. Res..

[73] Francoz D., Buczinski S., Bélanger A.M., Forté G., Labrecque O., Tremblay D., Wellemans V., Dubuc J. (2015). Respiratory Pathogens in Québec Dairy Calves and Their Relationship with Clinical Status, Lung Consolidation, and Average Daily Gain. J. Vet. Intern. Med..

[74] Ellis J. (2019). What is the evidence that bovine coronavirus is a biologically significant respiratory pathogen in cattle?. Can. Vet. J..

[75] Ohlson A., Heuer C., Lockhart C., Tråvén M., Emanuelson U., Alenius S. (2010). Risk factors for seropositivity to bovine coronavirus and bovine respiratory syncytial virus in dairy herds. Vet. Rec..

[76] Toftaker I., Sanchez J., Stokstad M., Nødtvedt A. (2016). Bovine respiratory syncytial virus and bovine coronavirus antibodies in bulk tank milk—Risk factors and spatial analysis. Prev. Vet. Med..

[77] Brscic M., Leruste H., Heutinck L.F.M., Bokkers E.A.M., Wolthuis-Fillerup M., Stockhofe N., Gottardo F., Lensink B.J., Cozzi G., Van Reenen C.G. (2012). Prevalence of respiratory disorders in veal calves and potential risk factors. J. Dairy Sci..

[78] Svensson C., Liberg P. (2006). The effect of group size on health and growth rate of Swedish dairy calves housed in pens with automatic milk-feeders. Prev. Vet. Med..

[79] Walter Swerczek T. (2019). Exacerbation of Streptococcus Equi (Strangles) by Overly Nutritious Diets in Horses: A Model for Infectious Bacterial Diseases of Horses and Other Livestock. Anim. Vet. Sci..

[80] Galyean M.L., Perino L.J., Duff G.C. (1999). Interaction of cattle health/immunity and nutrition2. J. Anim. Sci..

[81] Whitney T.R., Duff G.C., Collins J.K., Schafer D.W., Hallford D.M. (2006). Effects of diet for early-weaned crossbred beef steers on metabolic profiles and febrile response to an infectious bovine herpesvirus-1 challenge. Livest. Sci..

[82] Sharon K.P., Liang Y., Sanchez N.C.B., Carroll J.A., Broadway P.R., Davis E.M., Ballou M.A. (2019). Pre-weaning plane of nutrition and Mannheimia haemolytica dose influence inflammatory responses to a bovine herpesvirus-1 and Mannheimia haemolytica challenge in post-weaning Holstein calves. J. Dairy Sci..

